# Collection-based analysis of selected medical libraries in the Philippines using Doody’s Core Titles

**DOI:** 10.5195/jmla.2017.103

**Published:** 2017-01

**Authors:** Efren Torres

## Abstract

**Objectives:**

This study assessed the book collection of five selected medical libraries in the Philippines, based on Doodys’ Essential Purchase List for basic sciences and clinical medicine, to compare the match and non-match titles among libraries, to determine the strong and weak disciplines of each library, and to explore the factors that contributed to the percentage of match and non-match titles.

**Method:**

List checking was employed as the method of research.

**Results:**

Among the medical libraries, De La Salle Health Sciences Institute and University of Santo Tomas had the highest percentage of match titles, whereas Ateneo School of Medicine and Public Health had the lowest percentage of match titles. University of the Philippines Manila had the highest percentage of near-match titles.

**Conclusion:**

De La Salle Health Sciences Institute and University of Santo Tomas had sound medical collections based on Doody’s Core Titles. Collectively, the medical libraries shared common collection development priorities, as evidenced by similarities in strong areas. Library budget and the role of the library director in book selection were among the factors that could contribute to a high percentage of match titles.

## INTRODUCTION

Libraries typically aim to keep their collections fresh and updated based on client needs and professional standards. Aside from quantity, the quality of collections is constantly improved and maintained, because this is one of the fundamental services of libraries. High-quality collections also demonstrate the relevance and importance of libraries to the communities they serve. Thus, collection analysis is a significant aspect of library management.

List-checking is a method of collection analysis that librarians use to ensure the quality of resources. It establishes the authoritativeness and relevance of a collection. Faigel noted that it is the most popular method used to qualitatively evaluate collections [1]. Crawley-Low said that it reflects local needs of patrons and identifies strengths and weaknesses of a collection [2], and Bergen and Nemec believed that it can serve as a basis for collection assessment initiatives [3]. Johnson mentioned that it can develop a librarian’s subject expertise [4].

Shedlock and Walton described Doody’s Core Titles (DCT) in the health sciences as a guide to selecting core titles in health sciences literature that was established in 2004 in response to the cessation of the Brandon/Hill list [5], which previously was the authoritative selection guide for health sciences titles. In his review of DCT 2004, Spasser pointed out that while the list might not be perfect, its selection and rating criteria are noteworthy, and it is an essential collection development tool for the health sciences [6]. Fischer evaluated DCT and found it to be comprehensive and its selection committee well-represented by medical and allied health faculty and librarians [7].

DCT identifies certain titles as “Essential Purchases” that are important for a small library collection [8]. These titles are categorized by specialty and are selected by at least two librarians. The items on the Essential Purchase List can be considered to be a base collection as opposed to all selected titles, which would be considered a core collection. Lamb defined a base collection as recommended titles used to build a new collection [9]. Therefore, DCT is an important tool for collection analysis among medical libraries and information centers. It helps libraries identify the minimum requirements or standards when building their collections, especially when their budgets are very limited. It can also be used to measure the value of an existing collection against a set standard, to identify the strengths and weaknesses of a collection, and to compare collections across different medical libraries.

The author assessed the book collection of selected medical libraries in the Philippines based on DCT 2014 Essential Purchase Titles for basic sciences and clinical medicine. I compared the percentages of match and non-match titles among libraries and determined the strong and weak disciplines of each library. Furthermore, I explored the factors that can contribute to differences in the percentage of match and non-match among libraries.

## METHODS

This study was descriptive and employed list-checking against DCT 2014 Essential Purchase Titles for basic sciences and clinical medicine. The monograph collections of five medical libraries in the Philippines were included: Ateneo School of Medicine and Public Health Library (ASMPH), De La Salle Health Sciences Institute Romeo P. Ariniego MD Library (DLSHSI), University of East Ramon Magsaysay Memorial Medical Center Library (UERMM), University of the Philippines Manila Dr. Floerentino Herrera Medical Library (UPM), and University of Santo Tomas Health Sciences Library (UST). The libraries were chosen based on geography, the availability of an online public access catalog (OPAC), and their consent to answer the questionnaire on library profile.

DCT titles were exported to Microsoft Excel and searched against the OPAC of the five libraries. To check updates in their collections, I searched against their respective OPACs in intervals from June to September 2014. The same methodology of checking via OPAC was previously used by Smith [10], Nissonger and Meehan [11], and Meehan, Swanson, Yates, and Decker [12]. If a library had a book with the same title and edition as listed in the core list, it was recorded as an exact match. If a library had a book with the same but superseded title as that in the core list, it was a near-match. If a library did not have a title in the core list, it was a non-match. Exact and near-match titles were combined and considered match titles.

In the assessment of strong and weak subject areas, subjects consisting of less than three titles were excluded from the list. These subjects were: anatomy/embryology, biochemistry, immunology, microbiology, and neuroscience for the basic sciences; critical care, endocrinology, gastroenterology, health care administration, laboratory medicine, nephrology, ophthalmology, rheumatology, and urology for clinical medicine.

## RESULTS

### Exact and near-match titles among libraries

Table 1 shows the numbers and percentages of exact and near-match titles among libraries. Considering basic sciences titles, UST had the highest percentage of exact matches, whereas UPM had the lowest percentage of exact matches. However, UPM had the highest percentage of near-match titles. Considering clinical medicine titles, DLSHSI had the highest percentage of exact matches, whereas UERMM had the lowest percentage of exact matches. UST had the highest percentage of near-match titles. When basic sciences and clinical medicine titles were combined, DLSHSI had the highest percentage of exact matches, and UPM had the highest percentage of near-matches. Conversely, UPM had the lowest percentage of exact matches, and ASMPH had the lowest percentage of near-matches.

**Table 1:** Exact match and near-match titles EPL (basic sciences, n=45; clinical medicine, n=201)

	Basic sciences						Clinical medicine						Combined				
	Exact match		Near-match		Total match		Exact match		Near-match		Total match		Exact match		Near-match		Total
Library	n	%	n	%	n	%	n	%	n	%	n	%	n	%	n	%	n
ASMPH	22	48.89%	3	6.67%	25	55.56%	54	26.87%	9	4.48%	63	31.34%	76	86.36%	12	13.64%	88
DLSHSI	28	62.22%	5	11.11%	33	73.33%	132	65.67%	15	7.46%	147	73.13%	160	88.89%	20	11.11%	180
UERMM	20	44.44%	8	17.78%	28	62.22%	43	21.39%	45	22.39%	88	43.78%	63	54.31%	53	45.69%	116
UPM	12	26.67%	17	37.78%	29	64.44%	46	22.89%	57	28.36%	103	51.24%	58	43.94%	74	56.06%	132
UST	29	64.44%	7	15.56%	36	80.00%	70	34.83%	64	31.84%	134	66.67%	99	58.24%	71	41.76%	170

ASMPH=Ateneo School of Medicine and Public Health Library.

DLSHSI=De La Salle Health Sciences Institute Romeo P. Ariniego MD Library.

UERMM=University of East Ramon Magsaysay Memorial Medical Center Library.

UPM=University of the Philippines Manila Dr. Floerentino Herrera Medical Library.

UST=University of Santo Tomas Health Sciences Library.

### Match and non-match titles among libraries

Table 2 shows the numbers and percentages of match and non-match titles among libraries. When exact and near-match titles were combined to represent match titles, UST had the highest percentage of basic sciences titles, and DLSHSI had the highest percentage of clinical medicine titles. ASMPH had the lowest percentage of match titles for both basic sciences and clinical medicine. When basic sciences and clinical medicine titles were combined, DLSHSI had the lowest percentage of non-match titles, whereas ASMPH had the highest percentage of non-match titles.

**Table 2:** Match and non-match titles (n=246)

	Basic sciences		Match Clinical medicine		Combined		Basic sciences		Non-match Clinical medicine		Combined	
Library	n	%	n	%	n	%	n	%	n	%	n	%
ASMPH	25	55.56%	63	31.34%	88	35.77%	20	44.44%	138	68.66%	158	64.23%
DLSHSI	33	73.33%	147	73.13%	180	73.17%	12	26.67%	54	26.87%	66	26.83%
UERMM	28	62.22%	88	43.78%	116	47.15%	17	37.78%	113	56.22%	130	52.85%
UPM	29	64.44%	103	51.24%	132	53.66%	16	35.56%	98	48.76%	114	46.34%
UST	36	80.00%	134	66.67%	170	69.11%	9	20.00%	67	33.33%	76	30.89%

### Strong and weak subjects among libraries

Tables 3 and 4 present the strong and weak areas in basic sciences and clinical medicine titles, respectively, among libraries based on the percentage of match titles. Regarding basic sciences titles, most libraries were strong in physiology but weak in epidemiology and biostatistics. In terms of clinical medicine titles, most libraries were strong in dermatology but weak in other subjects. For most of the strong subjects, all DCT recommended titles (100%) were available in the libraries’ collections.

**Table 3:** Strong and weak subjects based on percentage of match titles for basic sciences

Subject	ASMPH	DLSHSI	UERMM	UPM	UST
Biostatistics	11%	33%	22%	22%	44%
Cell biology/histology	80%	90%	60%	60%	80%
Epidemiology	—	33%	—	67%	67%
Molecular biology	33%	67%	33%	100%	100%
Pathology	33%	67%	100%	100%	67%
Pharmacology	67%	67%	100%	100%	100%
Physiology	100%	100%	100%	75%	100%

**Table 4:**
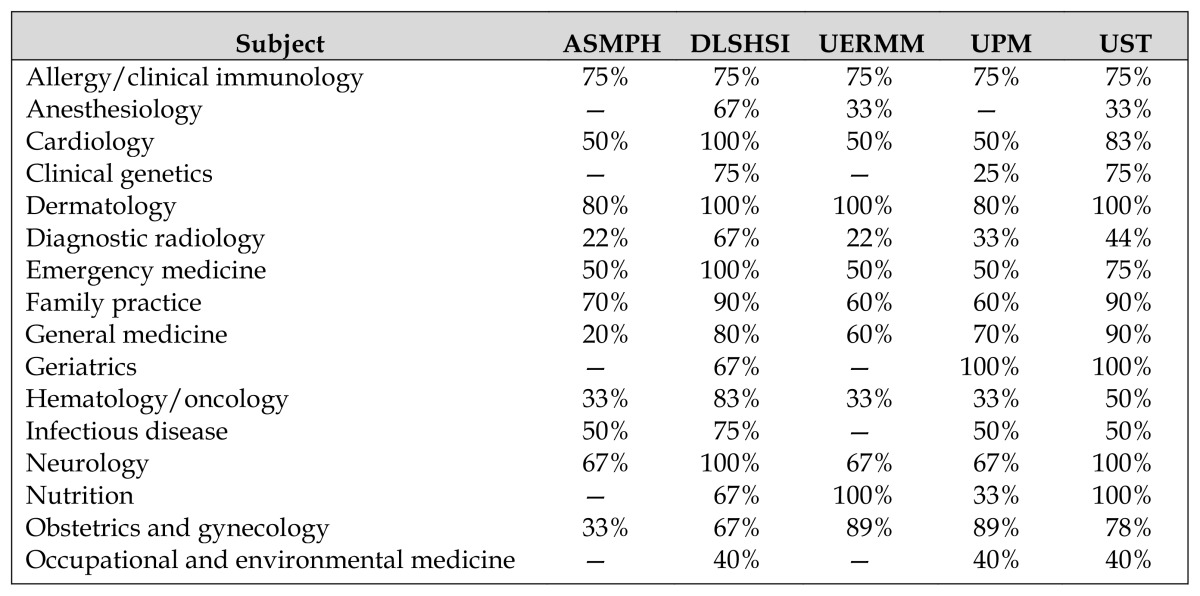
Strong and weak subjects based on percentage of match titles in clinical medicine

Subject	ASMPH	DLSHSI	UERMM	UPM	UST
Allergy/clinical immunology	75%	75%	75%	75%	75%
Anesthesiology	—	67%	33%	—	33%
Cardiology	50%	100%	50%	50%	83%
Clinical genetics	—	75%	—	25%	75%
Dermatology	80%	100%	100%	80%	100%
Diagnostic radiology	22%	67%	22%	33%	44%
Emergency medicine	50%	100%	50%	50%	75%
Family practice	70%	90%	60%	60%	90%
General medicine	20%	80%	60%	70%	90%
Geriatrics	—	67%	—	100%	100%
Hematology/oncology	33%	83%	33%	33%	50%
Infectious disease	50%	75%	—	50%	50%
Neurology	67%	100%	67%	67%	100%
Nutrition	—	67%	100%	33%	100%
Obstetrics and gynecology	33%	67%	89%	89%	78%
Occupational and environmental medicine	—	40%	—	40%	40%
Orthopedics	20%	100%	60%	80%	80%
Otolaryngology	60%	60%	80%	40%	40%
Pediatrics	50%	75%	88%	75%	75%
Physical medicine and rehabilitation	29%	100%	57%	71%	100%
Psychiatry	25%	25%	25%	25%	25%
Public health	25%	50%	50%	50%	100%
Pulmonology	20%	60%	60%	60%	20%
Radiation oncology	—	75%	13%	25%	63%
Sports medicine	20%	90%	30%	20%	80%
Surgical pathology	—	75%	25%	38%	63%
Surgery and related subspecialties	15%	52%	22%	37%	30%

### Relationship between library characteristics and percentage of match and non-match titles

Table 5 presents characteristics of the five libraries. UST and DLSHSI had the highest percentage of match titles as well as the largest book budgets. However, these two libraries differed in age, with UST being the oldest library and DLSHSI being the second youngest library. UPM had the highest percentage of near-match titles. This library was the second oldest library and had the largest book collection. ASMPH and UERMM ranked first and second, respectively, among libraries with the highest percentage of non-match titles for basic sciences, clinical medicine, and combined titles. These libraries had the smallest budgets, and ASMPH was the youngest library. The library directors of all libraries, except UERMM, had the authority to select books for their libraries. ASMPH, DLSHSI, and UPM used DCT as a selection tool. Rather than DCT, UST used National Library of Medicine and Brandon/Hill core lists, and UERMM selected books based on the prescribed textbooks of its faculty.

**Table 5:** Library profiles

	Age (years)	Library budget	Number of titles and volumes	Library book selector	Knowledge of DCT	Subscription to DCT	Use of DCT
ASMPH	7	Php 1–1.5M (USD $22,000–$33,000)	4,701 titles 5,618 volumes	Library director	Yes	No	Yes, via partner library
DLSHSI	30	Php 2.5–3M (USD $55,000–$67,000)	19,251 titles 25,148 volumes	Library director	Yes	Yes, since January 2012	Yes
UERMM	53	Php 1–1.5M (USD $22,000–$33,000)	14,475 titles 28,973 volumes	College dean, library committee	Yes	Yes, since July 2014	No
UPM	109	Php 1.5–2M (USD $33,000–$45,000)	46,030 volumes	Library director	Yes	Yes, since June 2011	Yes
UST	143	Php 2.5–3M (USD $55,000–$67,000)	20,904 titles 25,311 volumes	Library director, library coordinators	Yes	No	No

## DISCUSSION

I found that UST and DLSHSI had sound and good-quality collections with the highest percentages of matches in basic sciences, clinical medicine, and combined titles. UPM and UERMM had high percentages of near-matches, reflecting the obsolescence of their collections. ASMPH had the highest number of non-match titles, which suggests deficiencies in its collection.

All five libraries had comparable strong subjects in basic sciences and clinical medicine, which shows similarities in their collection priorities. However, the libraries had different weak areas. Due to budget limitations, it may difficult to develop all weak areas at once. Thus, one library might choose to develop one area, while other libraries might strengthen another area. These differences in weak areas could be considered a driving force for forming consortiums for collection sharing.

The age of a medical school can be either beneficial or detrimental to its library collection. Ideally, an older medical school has a head start in developing its collection, compared with a younger school that is still building its collection. UST, the oldest medical library, exhibited this effect and had a high percentage of match titles compared with other libraries. However, this may not always be the case, as age does not guarantee a high percentage of match titles. UPM and UERMM are older medical schools but had high percentages of non-match titles, compared with other libraries. On the contrary, DLSHSI had a high percentage of match titles compared with other libraries but is one of the youngest libraries. Thus, age can be a disadvantage when it leads to a high percentage of near-match titles and hence obsolescence. Older libraries have a tendency to keep superseded editions in their collections, as illustrated in the case of UPM, which had more near-match than exact match titles. On the other hand, younger libraries tend to only acquire the latest editions in their collections, as in the case of DLSHSI and ASMPH, which are younger libraries and had more exact matches.

Budget appears to be among the factors that contribute to a high percentage of match titles, as higher budgets give a library spending power to purchase books and keep its collection current. DLSHSI and UST had the highest percentage of match titles and had the largest budgets among the medical libraries. On the other hand, ASMPH and UERMM had more non-match than match titles and had smaller budgets than the other libraries.

The size of a collection can also contribute to a high percentage of match titles, because it increases the chance of having an exact or near-match title. However, similar to the age of a medical school, collection size could also contribute to the prevalence of more near-match than exact match titles, reflecting an obsolete collection. This was seen for UPM, which had a high percentage of match titles, although near-matches accounted for most of these titles. By contrast, ASMPH had the lowest percentage of match titles, but most of these were exact matches, making its collection relatively current.

The use of DCT as a selection tool as well as the library director’s role as a book selector may be additional factors that contribute to the percentage of match titles. DLSHSI and UPM shared these characteristics and had more match titles than UERMM, which did not employ DCT as a selection tool and whose library director was not involved in the selection process. Although UST did not subscribe to or use DCT, its age, budget, collection size, and the library director’s role in the selection process may have afforded it the opportunity to acquire more DCT-listed titles. It should also be noted that libraries that were aware of DCT might not necessarily have used it as a selection tool, as in the case of UST and UERMM. Furthermore, a library might subscribe to DCT but not use it as a selection tool, as observed for UERMM, or a library might use DCT as a selection tool but opt to partially follow the list, as observed for ASMPH.

### Limitations

This study has limitations that should be acknowledged. Even if a title was owned, it might not have been available during the period of evaluation. My findings represent a snapshot of a moment in time, thus holdings could be different if checked at a later date. Differences in library mission and financial or space limitations may be other factors influencing their collections, but these are beyond the scope of the study. For instance, the library or medical program might not be among the high-priority departments or academic programs of a university, which could result in a lower budget and minimal management support, thereby lowering the quality of the library collection. Space limitations may drive some libraries to acquire e-books and not catalog them in their OPACs, and thus these titles might not be accounted for when the OPAC is searched for match titles. Use of a larger checklist might have been desirable, especially since some of the subject areas were exceptionally small. Further studies could include checking the libraries’ collections against the entire DCT core titles list. A user-centered collection assessment technique could also be employed to provide useful feedback on the actual use and relevance of the collection.

## CONCLUSION

The findings demonstrate that medical libraries in the Philippines vary in terms of the quality of their collections. Some libraries have a sound collection, as evidenced by a high percentage of match titles. Other libraries lean toward obsolescence due to a high percentage of near-match titles. Still other libraries have poor collections with a high percentage of non-match titles. Many factors contribute to the percentage of match and non-match titles such as budget, the role of the library director as a selector, and the use of DCT as a selection tool. Libraries may consider reviewing their policies and practices to improve the quality of their collections, such as increasing the budget, involving the library director in the selection process, and using DCT as a benchmark for their collections. Medical libraries may share common collection development priorities but differ in areas that need to be improved. They are encouraged to consider their collection priorities to build their weak areas to achieve a balanced collection with all medical subjects and specialties fairly represented in their collections. A consortium for resource sharing or cooperative acquisition may help address gaps in library collections while managing costs.
